# Emotional responses to irony and emoticons in written language: Evidence from EDA and facial EMG

**DOI:** 10.1111/psyp.12642

**Published:** 2016-03-17

**Authors:** Dominic Thompson, Ian G. Mackenzie, Hartmut Leuthold, Ruth Filik

**Affiliations:** ^1^ School of EnglishUniversity of NottinghamNottinghamUK; ^2^ Psychological Institute, University of TübingenTübingenGermany; ^3^ School of PsychologyUniversity of NottinghamNottinghamUK

**Keywords:** Irony, Emoticons, Psychophysiology, Language

## Abstract

While the basic nature of irony is saying one thing and communicating the opposite, it may also serve additional social and emotional functions, such as projecting humor or anger. Emoticons often accompany irony in computer‐mediated communication, and have been suggested to increase enjoyment of communication. In the current study, we aimed to examine online emotional responses to ironic versus literal comments, and the influence of emoticons on this process. Participants read stories with a final comment that was either ironic or literal, praising or critical, and with or without an emoticon. We used psychophysiological measures to capture immediate emotional responses: electrodermal activity to directly measure arousal and facial electromyography to detect muscle movements indicative of emotional expressions. Results showed higher arousal, reduced frowning, and enhanced smiling for messages with rather than without an emoticon, suggesting that emoticons increase positive emotions. A tendency toward less negative responses (i.e., reduced frowning and enhanced smiling) for ironic than literal criticism, and less positive responses (i.e., enhanced frowning and reduced smiling) for ironic than literal praise suggests that irony weakens the emotional impact of a message. The present findings indicate the utility of a psychophysiological approach in studying online emotional responses to written language.

The basic function of irony is to communicate the opposite of what is said (Grice, 1975). This indirectness can make ironic language harder to understand than literal language and increases the chances of misinterpretation. However, the benefit of using irony comes from its various subtle and complex effects, serving additional communicative functions that would be absent in the literal equivalent. For example, if Paddy does something particularly clumsy, Sara may respond with **“**Nicely done!**”** in which case Sara may intend to elicit a certain emotional response, such as amusement or anger. Here, we will examine immediate emotional reactions to ironic and literal comments in written communication.

One controversial issue is whether using irony increases or decreases the positive or negative impact of a message. Brown and Levinson (1987) argued that one function of irony is to reduce threat. In line with this, Dews and Winner (1995) present evidence that irony reduces the strength of a statement: criticism becomes less negative, and praise becomes less positive (see also Harris & Pexman, 2003). Based on such findings, Dews and Winner proposed the tinge hypothesis, according to which the meaning of an ironic comment is automatically affected by the literal meaning. In ironic criticism, the intended disapproval of “Nicely done!” is automatically “tinged” with the positive literal reading of the phrase, and vice versa for ironic praise. The implication of the tinge hypothesis is that an ironic comment provokes a weaker emotional response in the recipient than its literal equivalent, and thus may have less impact on the relationship between speaker and recipient.

An alternative view follows the findings of Leggitt and Gibbs (2000). According to their rating studies, different forms of irony evoke different emotions. The negative emotions elicited most strongly by ironic criticism (or sarcasm) are anger, disgust, and contempt. Notably, they found that, in comparison to literal criticism, sarcasm is associated with a higher degree (greater arousal) of negative emotions. One explanation for this increase is that irony also conveys information about the speaker's attitude. It has been argued that irony is especially appropriate if the speaker wants to indicate a hostile attitude toward the recipient (Lee & Katz, 1998). Others agree that irony might enhance emotional responses, such as the emotions felt when experiencing criticism (Toplak & Katz, 2000) or condemnation (Colston & Gibbs, 2007). In recent work (Filik, Hunter, & Leuthold, 2015), the enhancing effect of irony has also been observed for praise. Thus, ironic comments may provoke stronger emotional responses than literal comments.

Correctly interpreting irony can be more difficult than interpreting literal language; hence, speakers may utilize paralinguistic features to clarify their intentions, such as tone of voice, facial expressions, and gestures. While irony is very frequently encountered in speech, it is also commonly experienced in writing (Hancock, 2004), where these cues are largely absent. Written instant communication is now extremely common; e‐mail, SMS, and other messaging services (e.g., WhatsApp, Viber) enable typewritten conversations to occur with a level of immediacy more comparable to spoken communication. Language users have developed various ways of compensating for the lack of paralinguistic features, most creatively by using emoticons. One of the primary uses for emoticons is to express emotion or attitude, but also to put a comment in perspective or strengthen a message (Derks, Bos, & von Grumbkow, 2008). It has also been suggested that using emoticons makes communication more enjoyable (Huang, Yen, & Zhang, 2008).

Corpus studies have shown that ironic messages in computer‐mediated communication are often accompanied by emoticons. Although there is no single meaning or emotion expressed by any given emoticon (Dresner & Herring, 2010), variants of the **;)** (wink‐face) and :**p** (tongue‐face) emoticons have been shown to frequently co‐occur with ironic statements (e.g., Carvalho, Sarmento, Silva, & De Oliveira, 2009; Derks, Bos, & von Grumbkow, 2007; Garrison, Remley, Thomas, & Wierszewski, 2011). These corpus observations are supported by rating studies (Filik et al., 2015) and by two large production studies reported in Thompson and Filik (in press), in which participants were specifically tasked with making their ironic intentions clear. The authors found that participants were significantly more likely to use variants of **;)** or :**p** than any other emoticon or textual device (such as LOL) when explicitly signaling ironic intent. The majority of emoticons have several variants; for example, the tongue‐face emoticon discussed above can be rendered as **:P, :‐p**, or **8‐p**, among others. Thompson and Filik report that participants show an overwhelming preference for the basic :**p** form.

Previous studies on the emotional impact of ironic language have usually asked participants to rate how a recipient would feel along a given dimension. Such a task highlights the emotional content of the materials and allows participants to think about, and potentially change, their responses. Thus, such “offline” tasks fail to capture the immediate emotional response to irony. In order to better capture moment‐to‐moment emotional responses to ironic language, we will use online psychophysiological measures. Electrodermal activity (EDA) will be recorded to measure tonic and phasic responses to emotionally arousing stimuli (Dawson, Schell, & Filion, 2007), and facial electromyography (EMG) will be used to detect facial muscle movement (Dimberg, 1990), indicating the presence and degree of certain emotional expressions (van Boxtel, 2010). Most important for present purposes are activity in the zygomaticus major (indicative of smiling) and the corrugator supercilii (frowning).

### Objectives and Hypotheses

Our aim is to reveal the emotional impact of ironic language compared to literal language in a variety of written contexts. Having participants read instances of criticism and praise, delivered both ironically and literally, will allow us to examine whether irony has a greater or lesser impact than a literal equivalent, as well as whether the effect differs according to message polarity. Here, we concentrate on irony in its most basic form, which entails saying one thing while meaning the opposite. Once the effects of basic irony are understood, future studies can examine more complex forms of irony, such as hyperbole, and the combination of irony with other linguistic devices, such as metaphor.

According to the tinge hypothesis (Dews & Winner, 1995), irony weakens emotional impact; hence, we would predict a reduced EDA response for ironic compared to literal comments. We would also expect to observe less activity in the zygomaticus major (i.e., reduced smiling) when praise is delivered ironically rather than literally, and less activity in the corrugator supercilii (i.e., reduced frowning) for criticism that is delivered ironically rather than literally. By contrast, if irony enhances emotional impact (Leggitt & Gibbs, 2000), we would expect the reverse.

We will also examine the effect of including an irony‐appropriate emoticon. We selected the :**p** (tongue‐face) emoticon for several reasons. As noted above, the tongue face is observed alongside ironic comments in several corpus studies (e.g., Carvalho et al., 2009), and has been shown to be used to explicitly mark irony (Thompson & Filik, in press). Although the **;)** wink‐face emoticon is also frequently used to mark irony, we chose the :**p** tongue‐face since it does not include a “smile” element, which participants could potentially mimic. While there are several variants of the tongue‐face emoticon, we selected the most frequently used form, :**p**.

It has been suggested that smiling emoticons increase message positivity and frowning ones increase negativity (Derks et al., 2007), though it is not clear what effect emoticons may have on the emotions elicited by irony. If, in general, emoticons increase the enjoyment of written communication (Huang et al., 2008), we would expect to see a global increase in smiling and decrease in frowning, as well as an increase in EDA response, when an emoticon is present rather than absent.

## Method

### Participants

Power analysis was conducted (using G*Power 3; Faul, Erdfelder, Lang, & Buchner, 2007) to estimate the sample size needed to achieve a statistical power of (1 − b) = .80, as recommended by Cohen (1988). Based on lowest effect size measures (partial eta‐squared) reported by previous studies analyzing facial EMG responses to language stimuli (e.g., Foroni & Semin, 2009), effect size was set to *f* = 0.22. With the significance level set to alpha = .05, the power analysis showed that at least 43 participants were needed. Fifty‐three native‐English speakers (mean age 24; 19 male) from the University of Nottingham population were recruited. Six showed no EDA response (nonresponders) and were excluded; hence, 47 were entered into the analyses (mean age 24; 16 male).

### Materials and Design

Two of the authors (both native speakers of English) generated the experimental materials. Materials were only included when both authors agreed that they followed the structure described below and sounded natural.^1^ One hundred and sixty materials were created, each consisting of two sentences (see Table 1 for an example and Appendix for a wider selection). Though the contexts and situations differed, each item followed the same basic structure: the first sentence provided a contextual setup, describing an event in which one person (the recipient) has done something either praiseworthy or criticismworthy, thereby manipulating the variable polarity (praise vs. criticism).

**Table 1:** Example of Materials Used, Each with Eight Conditional Variants

Conditional manipulations			Sentences
Criticism	Ironic	Emoticon	Susie texted Linda to say that she hadn't been to the gym at all that week. Linda texted her back to say: You're so motivated :**p**
		Full stop	Susie texted Linda to say that she hadn't been to the gym at all that week. Linda texted her back to say: You're so motivated.
	Literal	Emoticon	Susie texted Linda to say that she hadn't been to the gym at all that week. Linda texted her back to say: You're so unmotivated :**p**
		Full stop	Susie texted Linda to say that she hadn't been to the gym at all that week. Linda texted her back to say: You're so unmotivated.
Praise	Ironic	Emoticon	Susie texted Linda to say that she had been to the gym every day that week. Linda texted her back to say: You're so unmotivated :**p**
		Full stop	Susie texted Linda to say that she had been to the gym every day that week. Linda texted her back to say: You're so unmotivated.
	Literal	Emoticon	Susie texted Linda to say that she had been to the gym every day that week. Linda texted her back to say: You're so motivated :**p**
		Full stop	Susie texted Linda to say that she had been to the gym every day that week. Linda texted her back to say: You're so motivated.

The second sentence in each item described a second person (the speaker) responding to this event with a comment delivered via a written medium (text message, e‐mail, etc.). The content of this comment was either ironic or literal, reflecting the manipulation of the variable literality (ironic vs. literal). Materials were designed such that they would be disambiguated as being either literal or ironic when participants encountered the final word, allowing us to identify precisely when an emotional response would be expected to occur.^2^

The message also ended either with a full stop (.) or with an emoticon (**:p**), reflecting the variable emoticon (present vs. absent). This resulted in a 2 Polarity (praise vs. criticism) × 2 Literality (literal vs. ironic) × 2 Emoticon (present vs. absent) within‐subject design.

The 160 items each had eight conditional variants as described above and exemplified in Table 1. Items were assigned to eight counterbalanced lists: every item appeared exactly once per list and in a different condition in each of the eight lists, using a Latin square. This ensured an equal frequency of each condition in each list, such that participants would always see 20 items per condition, for a total of 160 experimental trials per list. The 160 items in each list were presented to participants in a randomized order. No filler materials were included, since neither EDA nor EMG is consciously controlled, and, hence, even if participants were to guess the aim of the study, they could not strategize in any way.

### Procedure

Participants were seated in a quiet lab room, in front of a 17‐inch computer monitor at a viewing distance of 70 cm. They used their dominant hand to control the computer keyboard, while their nondominant hand (with attached EDA electrodes) rested on a cushion on their lap.

**Figure 1:**
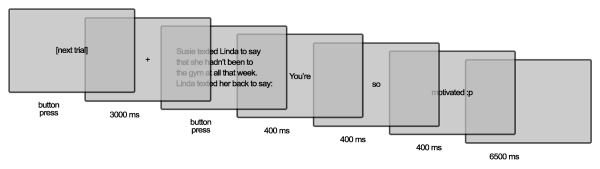
Example of trial procedure.

Trials proceeded as shown in Figure 1. The first screen displayed a message saying “[next trial].” Once participants pressed the space bar, a fixation cross appeared. This was displayed for 3,000 ms, after which the next screen appeared, showing the contextual setup sentence and the initial part of the second sentence, excluding the final comment. Participants read at their own pace and pressed the space bar when they had finished reading. The final sentence was then presented automatically, word by word, with each word displayed for 400 ms at the center of the screen. This allowed analysis of the psychophysiological recordings to be time‐locked to the onset of the final, disambiguating word. The full stop or emoticon appeared in conjunction with the final word.

The experiment was divided into four blocks with 40 items in each, allowing participants to take breaks as needed. Since participants were asked to read the materials silently, they could conceivably pretend to be reading. To prevent this, they answered comprehension questions at the end of each block to ensure they were processing the content of the materials. We took a correct response rate higher than 75% for each individual participant as indicating that they were properly attending to the content of the materials; all participants were above this threshold, and the average correct response rate was 90%.

### Psychophysiological Recording

A BioSemi Active‐Two amplifier system was used for the continuous recording of EMG and EDA signals; the latter signal was measured using an AC (16 Hz) constant current source with 1 µA amplitude. All signals were sampled at a frequency of 2048 Hz. Prior to electrode application, participants cleaned their hands using pH‐neutral hand wash. The EMG electrode positions were cleaned using alcohol pads (70%). Highly conductive saline electrode gel was used on the electrodes (SignaGel, Parker Laboratories, Fairfield, NJ). For the EDA recording, two flat Nihon Kohden Ag/AgCl electrodes (contact area diameter: 8 mm) were placed at the distal phalanges of the index and the middle fingers of the nondominant hand. For the EMG recording, pairs of Ag/AgCl electrodes (contact area diameter: 4 mm) were placed approximately 12 mm apart (center to center) over the two facial muscle regions of interest (left cheek and left eyebrow; cf. Fridlund & Cacioppo, 1986), that is, over the zygomaticus major (cheek) and corrugator supercilii (eyebrow).

### Data Analysis

Data preprocessing and analyses were carried out using MATLAB (Version 8.4.0) and available MATLAB toolboxes (Ledalab Version 3.46, Benedek & Kaernbach, 2010; available from www.ledalab.de; FieldTrip, Oostenveld, Fries, Maris, & Schoffelen, 2011) as well as custom MATLAB scripts.

As recommended by van Boxtel (2010), facial EMG data were first band‐pass filtered (20–500 Hz, 36 dB/octave), then rectified. The EMG data were time‐locked to the onset of the critical word with the analysis epoch extending 2,000 ms before and 5,000 ms after the critical word. Trials with extreme EMG values (>250 µV) were eliminated from the analysis. This resulted in a total data loss of ∼5.7% of trials (*N* = 427). The EMG amplitude was determined in 10 consecutive 400‐ms time intervals and expressed as a percentage of baseline EMG activity within the interval from −400 to 0 ms relative to critical word onset.

Skin conductance signals were downsampled to 16 Hz and then analyzed with the Ledalab toolbox using continuous decomposition analysis (CDA; Benedek & Kaernbach, 2010). CDA decomposes the skin conductance data into its constituent tonic and phasic components. More specifically, CDA yields the skin conductance level (SCL) as a continuous measure of tonic EDA, and the phasic driver underlying skin conductance data as a continuous measure of phasic EDA (cf. Benedek & Kaernbach, 2010). The phasic component represents the skin conductance response (SCR), which is an indicator of event‐related sympathetic activity.

Event‐related changes in skin conductance were determined for a response window from 1,000 to 6,000 ms after critical (final) word onset, that is, the average phasic driver activity (average SCR) within this time window as well as maximal SCR amplitude. A minimum amplitude criterion of 0.01 µS was applied. SCR data were checked for artifacts (e.g., extreme values), and epochs with artifacts were removed. This resulted in a total data loss of 1.53% and 0.43% of the observations (*N* = 115 and 32 of 7,520 observations) for average SCR and maximal SCR amplitude, respectively.

## Results

**Figure 2:**
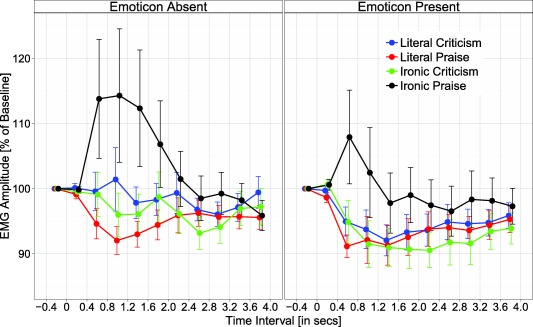
EMG corrugator response as a function of emoticon (absent vs. present), literality (literal vs. ironic), polarity (praise vs. criticism), and time window. Error bars reflect standard error of the mean.

EDA and EMG data were submitted to ANOVAs with repeated measures on the variables polarity (praise vs. criticism), literality (literal vs. ironic), and emoticon (absent vs. present). The EMG analysis additionally included the variable time window (10 levels: 0–400, 400–800, …, 3,600–4,000 ms). As can be seen in Figure 2, EMG corrugator activity generally decreased over time, an effect reflecting the fact that, during reading, participants contract their frowning muscles (indicating focus) and then relax these muscles when they have finished reading; this means participants are frowning at baseline. Therefore, when we refer to one condition having reduced frowning versus another, we are referring to a greater reduction from baseline.

### EDA

EDA data in the 5,000‐ms response window showed a larger amplitude when an emoticon was present rather than absent, significantly so in maximal SCR (0.623 vs. 0.586 µS), *F*(1,46) = 6.85, *p* < .05, η_p_^2^ = .13, and approaching significance in average SCR (0.305 vs. 0.292 µS), *F*(1,46) = 3.89, *p* = .054, η_p_^2^ = .08. No other effects were significant, all *F*s < 1.5, *p*s > .23.

### EMG

**Figure 3:**
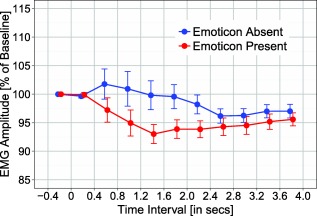
EMG corrugator response as a function of emoticon (absent vs. present) and time window. Error bars reflect standard error of the mean.

Grand mean EMG corrugator activity is depicted in Figure 2 as a function of experimental conditions and time window. It is evident that experimental conditions influenced EMG corrugator activity, and mainly so between 400 and 2,000 ms. Analysis of corrugator activity revealed a significant main effect of emoticon, *F*(1,46) = 7.65, *p* < .01, η_p_^2^ = .14, showing a larger reduction in activity (i.e., reduced frowning) for materials with an emoticon rather than without an emoticon (*M* = 95.4% vs. 98.64%). There was a significant Emoticon × Time Window interaction (see Figure 3), *F*(9,414) = 3.36, *p* < .05, ε = .42, η_p_^2^ = .07; the emoticon effect was significant between 1,200–1,600 ms, *t*(46) = 3.19, *p* = .003, and as a trend for time intervals 800–1,200 ms and 1,600–2,000 ms, *t*s(46) ≥ 2.75, *p*s ≤ .009 (Bonferroni‐corrected *α* level). The Literality × Time Window and the Polarity × Time Window interactions were significant, *F*(9,414) = 3.33, *p* < .05, ε = .28, η_p_^2^ = .07 and *F*(9,414) = 2.53, *p* < .05, ε = .408, η_p_^2^ = .05, respectively; however, *t* tests for individual time windows showed no reliable literality or polarity effects, all *t*s(46) < 1.95, *p*s > .057.

**Figure 4:**
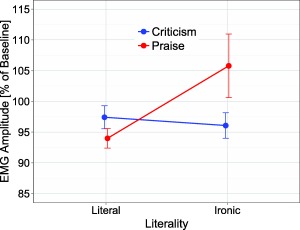
EMG corrugator response as a function of literality (literal vs. ironic) and polarity (praise vs. criticism). Error bars reflect standard error of the mean.

The Literality × Polarity interaction, *F*(1,46) = 5.87, *p* < .05, η_p_^2^ = .11, and the three‐way interaction of Literality × Polarity × Time Window were significant, *F*(9,414) = 3.40, *p* < .05, ε = .23, η_p_^2^ = .07. As can be seen in Figure 2, experimental effects were most pronounced during the time interval 400–2,000 ms (cf. Figure 3). Analysis of mean EMG amplitude for this time interval (400–2,000 ms) revealed significantly greater corrugator activity for ironic praise versus literal praise (105.43% vs. 93.99%), *t*(46) = 2.28, *p* = .027, but not for literal criticism versus ironic criticism (97.14% vs. 95.80%), *t*(46) = 0.77, *p* = .45; greater corrugator activity was also observed for ironic praise versus ironic criticism (105.43% vs. 95.80%), *t*(46) = 2.28, *p* = .027, and for literal criticism versus literal praise (97.14% vs. 93.99%), *t*(46) = 2.66, *p* = .011 (cf. Figure 4). No other effects approached significance, all *F*s < 2.64, *p*s > .11.

**Figure 5:**
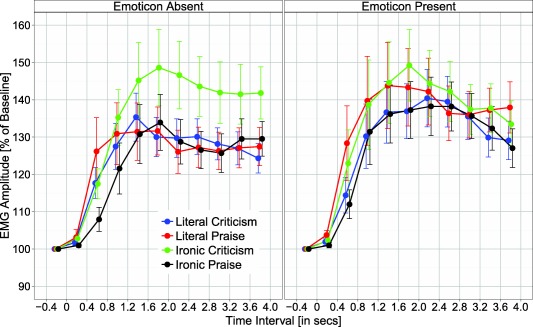
EMG zygomaticus response as a function of emoticon (absent vs. present), literality (literal vs. ironic), polarity (praise vs. criticism), and time window. Error bars reflect standard error of the mean.

Grand mean EMG zygomaticus activity is depicted in Figure 5 as a function of experimental conditions and time window. Analysis of zygomaticus activity revealed a main effect of emoticon, *F*(1,46) = 4.27, *p* < .05, η_p_^2^ = .08, this time indicating larger EMG activity (i.e., enhanced smiling) for materials with than without an emoticon (*M* = 131.19% vs. 126.69%). There was a main effect of time window, *F*(9,414) = 13.59, *p* < .001, ε = .26, η_p_^2^ = 0.23, indicating an initial increase and late decrease of EMG activity.

**Figure 6:**
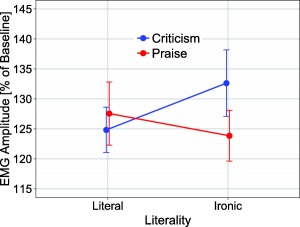
EMG zygomaticus response as a function of literality (literal vs. ironic) and polarity (praise vs. criticism). Error bars reflect standard error of the mean.

**Figure 7:**
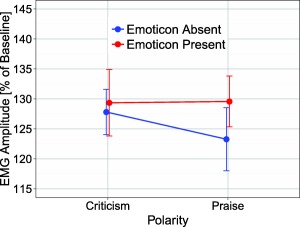
EMG zygomaticus response as a function of emoticon (absent vs. present) and polarity (praise vs. criticism). Error bars reflect standard error of the mean.

The Literality × Polarity interaction was significant, *F*(1,46) = 4.38, *p* < .05, η_p_^2^ = 0.09, reflecting reliably larger zygomaticus activity for ironic criticism than ironic praise (134.21% vs. 126.25%), *t*(46) = 2.97, *p* = .005, but only numerically larger activity for literal praise than literal criticism (129.88% vs. 126.25%), *t*(46) = 0.89, *p* = .38 (see Figure 6). There was also numerically larger zygomaticus activity for ironic criticism than literal criticism (134.21% vs. 126.25%), *t*(46) = 1.84, *p* = .07, and a reverse nonsignificant pattern for ironic praise versus literal praise (125.43% vs. 129.88%), *t*(46) = 0.91, *p* = .37. The Emoticon × Polarity interaction was significant, *F*(1,46) = 5.27, *p* < .05, η_p_^2^ = .10, indicating significantly reduced smiling for praise when an emoticon was absent rather than present (123.74% vs. 131.56%), *t*(46) = 2.79, *p* = .008, but no such emoticon effect for criticism (129.64% vs. 130.82%), *t*(46) = 0.56, *p* = .57 (see Figure 7). No other effects were significant (all *F*s < 2.28, *p*s > .08).

## Discussion

The present study examined participants' implicit emotional responses to written comments that were either ironic or literal, critical or praising, and either included or did not include an emoticon. There were a number of key findings. Notably, there were several robust effects of emoticon presence. EDA results indicated a higher level of arousal when an emoticon was present rather than absent. This effect was complemented by the EMG data, which showed a decrease in frowning activity and a complementary increase in smiling when an emoticon was present. Importantly, the emoticon we used had no “smile” element, meaning these observations did not arise due to a simple imitation effect. In line with earlier research (Huang et al., 2008), our data suggest that emoticons increase enjoyment in communication. The current findings also reveal that emoticons can effectively elicit positive emotions. Note, however, that we looked at just one emoticon. Further research should examine whether this effect extends to emoticons more generally.

In terms of the influence of irony on the emotional impact of criticism and praise, facial EMG data showed reduced frowning complemented by enhanced smiling for ironic compared to literal criticism, indicating that ironic criticism provoked a less negative response than literal criticism. There was also numerically enhanced frowning, complemented by reduced smiling for ironic compared to literal praise, indicating a less positive response to ironic than literal praise. This apparent weakening of emotional responses—the combination of irony making criticism less negative, and praise less positive—is in line with the tinge hypothesis (Dews & Winner, 1995), which claims that irony reduces the strength of a statement. However, while suggestive, it must be noted that only some of these patterns reached significance.

Facial EMG results also showed reliably enhanced frowning and less smiling for ironic praise compared to ironic criticism. This is perhaps surprising given that, intuitively, praise should not evoke a more negative response than criticism. This finding thus may instead reflect the fact that, in comparison to ironic criticism, ironic praise can be hard to understand (Harris & Pexman, 2003). Ironic praise is certainly less common than ironic criticism (Gibbs, 2000), and often has greater contextual dependencies. Interestingly, however, there was evidence of greater smiling for sentences conveying praise when an emoticon was present rather than absent. This may suggest that emoticons have an important function in clarifying intent. If this is the case for ironic praise, it is likely to extend to other contexts where interpretation is more difficult than usual—a question worth pursuing in future work.

In conclusion, we see that emoticons elicit positive emotion and heighten arousal, as well as serving to clarify intentions in more difficult contexts, highlighting their utility in relation to both modulating the emotional impact of a message and potentially aiding comprehension. There is also some evidence that irony reduces the strength of a message, making it less polarized. Thus, our findings contribute to the understanding of the moment‐to‐moment emotional effects of irony and the influence that emoticons have on this process.

## Footnotes

1 In the case of disagreements, revisions were made until both authors reached agreement; if no agreement could be met for a given material, it was rejected and another created in its place.

2 In order to isolate and investigate our specific factors of interest, we followed the standard psycholinguistic practice of constructing materials in which all other factors are equal. This would not be possible with “real‐world” utterances or corpus excerpts, since the materials would be too variable (e.g., overly diverse contexts, unknown/unclear contexts, no clear recipient of some comments).

## Appendix

**Table 1A:** Additional Examples of Materials Used, Each with Eight Conditional Variants

Item	Conditional manipulations			Sentences
1	Criticism	Ironic	Emoticon	Ella had been ill for a couple of days, and as usual James did nothing to assist, not even taking some of Ella's chores. She texted him to say: You're always so helpful :**p**
			Full stop	Ella had been ill for a couple of days, and as usual James did nothing to assist, not even taking some of Ella's chores. She texted him to say: You're always so helpful.
		Literal	Emoticon	Ella had been ill for a couple of days, and as usual James did nothing to assist, not even taking some of Ella's chores. She texted him to say: You're always so unhelpful :**p**
			Full stop	Ella had been ill for a couple of days, and as usual James did nothing to assist, not even taking some of Ella's chores. She texted him to say: You're always so unhelpful.
	Praise	Ironic	Emoticon	Ella had been ill for a couple of days, and as usual James did everything he could to assist, even taking all of Ella's chores and driving all the way to her office to drop off a report that was due. She texted him to say: You're always so unhelpful :**p**
			Full stop	Ella had been ill for a couple of days, and as usual James did everything he could to assist, even taking all of Ella's chores and driving all the way to her office to drop off a report that was due. She texted him to say: You're always so unhelpful.
		Literal	Emoticon	Ella had been ill for a couple of days, and as usual James did everything he could to assist, even taking all of Ella's chores and driving all the way to her office to drop off a report that was due. She texted him to say: You're always so helpful :**p**
			Full stop	Ella had been ill for a couple of days, and as usual James did everything he could to assist, even taking all of Ella's chores and driving all the way to her office to drop off a report that was due. She texted him to say: You're always so helpful.
2	Criticism	Ironic	Emoticon	Bethan noticed that her flatmate Helen always left the bath really dirty. She texted her to say: You always leave the place looking great :**p**
			Full stop	Bethan noticed that her flatmate Helen always left the bath really dirty. She texted her to say: You always leave the place looking great.
		Literal	Emoticon	Bethan noticed that her flatmate Helen always left the bath really dirty. She texted her to say: You always leave the place looking awful :**p**
			Full stop	Bethan noticed that her flatmate Helen always left the bath really dirty. She texted her to say: You always leave the place looking awful.
	Praise	Ironic	Emoticon	Bethan noticed that her flatmate Helen always left the bath sparkling clean. She texted her to say: You always leave the place looking awful :**p**
			Full stop	Bethan noticed that her flatmate Helen always left the bath sparkling clean. She texted her to say: You always leave the place looking awful.
		Literal	Emoticon	Bethan noticed that her flatmate Helen always left the bath sparkling clean. She texted her to say: You always leave the place looking great :**p**
			Full stop	Bethan noticed that her flatmate Helen always left the bath sparkling clean. She texted her to say: You always leave the place looking great.
3	Criticism	Ironic	Emoticon	Becca walked into Lara's office and saw that it was very messy and really quite unpleasant to be in. Becca emailed her later on saying: Your office is so lovely :**p**
			Full stop	Becca walked into Lara's office and saw that it was very messy and really quite unpleasant to be in. Becca emailed her later on saying: Your office is so lovely.
		Literal	Emoticon	Becca walked into Lara's office and saw that it was very messy and really quite unpleasant to be in. Becca emailed her later on saying: Your office is so horrible :**p**
			Full stop	Becca walked into Lara's office and saw that it was very messy and really quite unpleasant to be in. Becca emailed her later on saying: Your office is so horrible.
	Praise	Ironic	Emoticon	Becca walked into Lara's office and saw that it was very neat and really quite pleasant to be in. Becca emailed her later on saying: Your office is so horrible :**p**
			Full stop	Becca walked into Lara's office and saw that it was very neat and really quite pleasant to be in. Becca emailed her later on saying: Your office is so horrible.
		Literal	Emoticon	Becca walked into Lara's office and saw that it was very neat and really quite pleasant to be in. Becca emailed her later on saying: Your office is so lovely :**p**
			Full stop	Becca walked into Lara's office and saw that it was very neat and really quite pleasant to be in. Becca emailed her later on saying: Your office is so lovely.
4	Criticism	Ironic	Emoticon	After Bobby and Julia climbed just the few small steps up to the monument, Bobby was completely out of breath. Later Julia texted him saying: You are so fit :**p**
			Full stop	After Bobby and Julia climbed just the few small steps up to the monument, Bobby was completely out of breath. Later Julia texted him saying: You are so fit.
		Literal	Emoticon	After Bobby and Julia climbed just the few small steps up to the monument, Bobby was completely out of breath. Later Julia texted him saying: You are so unfit :**p**
			Full stop	After Bobby and Julia climbed just the few small steps up to the monument, Bobby was completely out of breath. Later Julia texted him saying: You are so unfit.
	Praise	Ironic	Emoticon	After Bobby and Julia climbed the hundreds of steps up to the monument, Bobby was not even slightly out of breath. Later Julia texted him saying: You are so unfit :**p**
			Full stop	After Bobby and Julia climbed the hundreds of steps up to the monument, Bobby was not even slightly out of breath. Later Julia texted him saying: You are so unfit.
		Literal	Emoticon	After Bobby and Julia climbed the hundreds of steps up to the monument, Bobby was not even slightly out of breath. Later Julia texted him saying: You are so fit :**p**
			Full stop	After Bobby and Julia climbed the hundreds of steps up to the monument, Bobby was not even slightly out of breath. Later Julia texted him saying: You are so fit.
